# The association between poor dental health and gastric cancer risk: a nationwide cohort and sibling-controlled study

**DOI:** 10.1186/s12916-025-04273-x

**Published:** 2025-07-21

**Authors:** Zengliang Ruan, Jianfeng Xie, Jingru Yu, Li Yin, Dariush Nasrollahzadeh Nesheli, Weimin Ye

**Affiliations:** 1 Key Laboratory of Environmental Medicine and Engineering of Ministry of Education, and Department of Epidemiology & Biostatistics, School of Public Health, Southeast University, Nanjing, China; 2 Department of Medical Epidemiology and Biostatistics, Karolinska Institutet, Nobels Väg 12a, 17165 Solna, Stockholm, Sweden; 3 AIDS/STD Prevention and Treatment Institute, Fujian Provincial Center for Disease Control and Prevention, Fuzhou, China; 4 Department of Epidemiology and Health Statistics, School of Public Health, Fujian Medical University, Fuzhou, China

**Keywords:** Gastric cancer, Dental health, Population-based, Register-based, Sibling-controlled, Cohort study

## Abstract

**Background:**

Poor dental health has been linked to an increased risk of gastric cancer (GC), but previous studies were limited by their retrospective design and relatively small sample size.

**Methods:**

We followed a nationwide cohort of 5,888,034 Swedish adults over the age of 19 who visited a dentist between 2009 and 2016. Additionally, a nested case-control study was conducted by comparing incident GC cases to their siblings. Cox regression analyses, using attained age as the timescale and adjusting for potential confounders, were performed to evaluate the association between various dental health conditions and the risk of GC. In addition, we stratified our analyses by sex and age and conducted various sensitivity analyses to ensure the robustness of our findings.

**Results:**

Over an average follow-up of 6.4 years, we identified 3993 new GC cases, including 1241 cardia GC and 2752 non-cardia GC. Compared to individuals with healthy teeth, those with periodontitis had an 11% and 25% increased risk of GC and cardia GC, respectively. The positive associations between odontogenic inflammation and the risk of GC were consistent in sibling-controlled analyses. We also observed a dose-response relationship between the number of remaining teeth and the risk of GC, with fewer teeth associated with higher risks. Additionally, we did not find significant interactions between dental inflammatory conditions and the number of remaining teeth in relation to the risk of GC or its subtypes. Our findings were consistent across different sex and age subgroups and in sensitivity analyses.

**Conclusions:**

This study provides the largest prospective cohort study evidence to date, along with the first sibling-controlled comparisons, supporting the association between poor dental health and GC risk. Promoting dental health in the general population could have significant public health implications in preventing this disease.

**Supplementary Information:**

The online version contains supplementary material available at 10.1186/s12916-025-04273-x.

## Background

Gastric cancer (GC), which originates from the epithelial cells of the gastric mucosa, is a malignancy associated with poor overall survival rates [1, 2]. Based on its anatomical location, GC can be classified as cardia cancer or non-cardia gastric cancer [3]. Despite a decline in recent decades, GC remains the fifth most common cancer and the fourth leading cause of cancer-related deaths worldwide, posing a significant global health burden [4, 5]. In 2020, there were an estimated 1.09 million new cases and 770,000 deaths from GC globally, corresponding to age-standardized incidence and mortality rates of 11.1 and 7.7 per 100,000 person-years, respectively [3, 4]. Therefore, it is crucial to understand the risk factors for GC, in order to develop effective public health interventions and prevention strategies.

Dental health problems include conditions affecting the teeth and surrounding tissues, such as periodontal disease, gingival inflammation, gum bleeding, tooth decay, and tooth loss [6, 7]. A complex interaction between oral microbiota, host immune susceptibility, and environmental factors may lead to pathogenesis of odontogenic diseases [8, 9]. Previous research has suggested a link between odontogenic disease and systematic conditions such as cancers, particularly those of the digestive system, including oral, pancreatic, esophageal, liver, and gastric cancers [7, 10, 11]. However, the relationship between specific dental health condition and GC remains controversial [12]. For example, a bidirectional Mendelian randomization study found no causal relationship between tooth loss or periodontal disease and GC [13]. Additionally, a recent meta-analysis concluded that periodontitis is associated with an increased risk of GC, while other dental problems such as denture wearing, gingivitis, tooth loss, and tooth brushing are not [12]. This inconsistency might be due to differences in study populations, exposure measurements, adjusted variables, or insufficient sample sizes. To address these limitations, further large-scale, high-quality prospective studies are needed to examine various dental health conditions and their potential causal relationship with GC.

Studies examining the relationship between dental health and gastrointestinal malignancies traditionally focus on tooth count, as it is simpler to measure than assessing inflammation or grading its severity in odontogenic diseases [14, 15]. This study used Swedish nationwide registers to explore the impact of both tooth count and dental health problems on the long-term risk of GC, including its cardia and non-cardia subtypes, in Swedish adults aged 19 and older. Additionally, sibling comparisons were also conducted in our study, which can provide the most direct evidence of the association between dental health and GC through effectively reducing the potential biases arising from familial confounders and other unmeasured factors shared by siblings, including both genetic and environmental influences [16].

## Methods

### Cohort design and study population

**Fig. 1:**
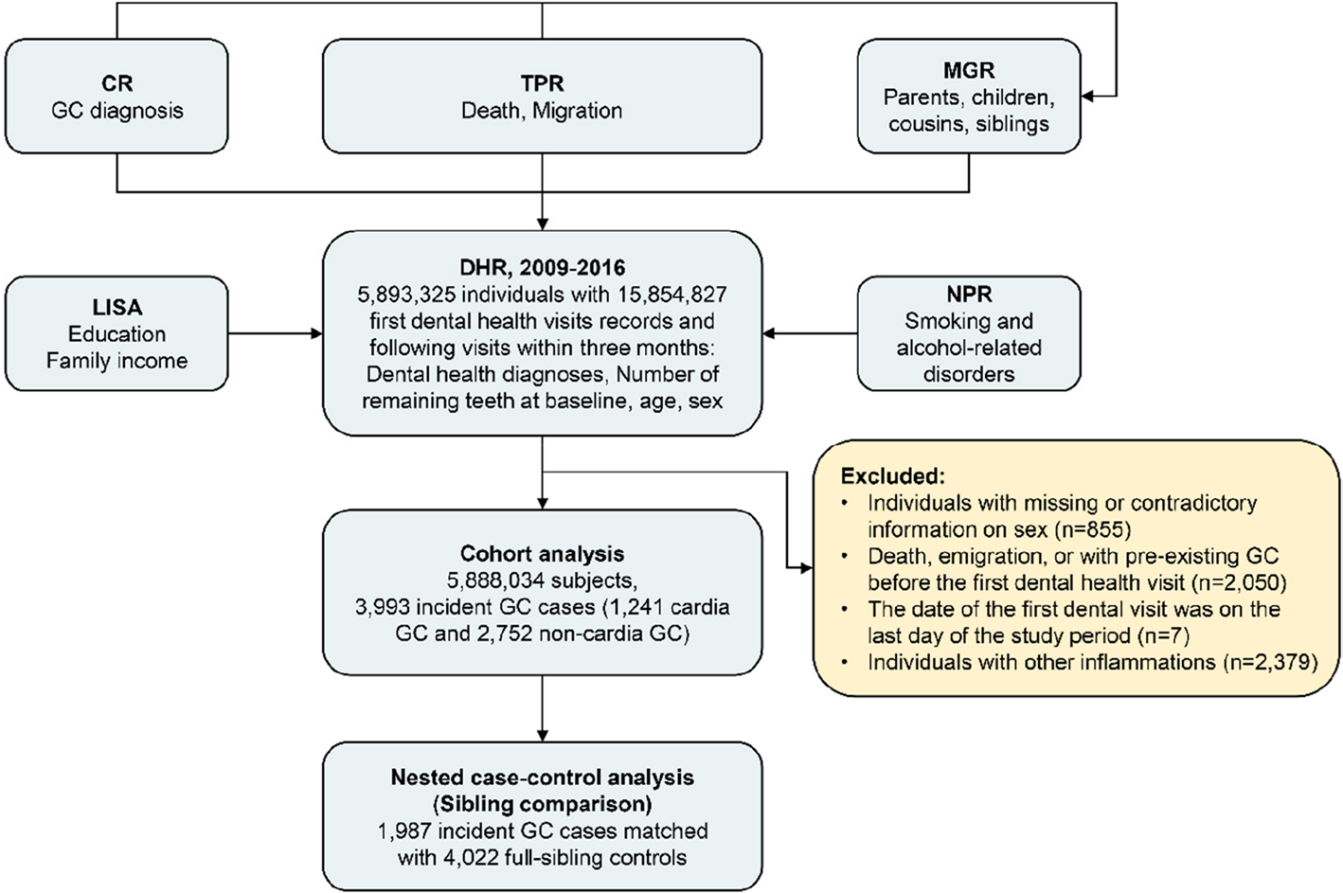
Flowchart of the cohort assembly process. Abbreviations: CR, the Swedish Cancer Register; GC, gastric cancer; TPR, the Swedish Total Population Register; NPR, the Swedish National Patient Register; LISA, the longitudinal integrated database for health insurance and labour market studies; DHR, the Swedish Dental Health Register; MGR, the Swedish Multi-Generation Register

Based on the population-based Dental Health Register (DHR), which contains nationwide coverage of individual data on dental health care [17], this prospective cohort study included Swedish adults aged over 19 years who visited a dentist at least once between 2009 and 2016. During the study period, we identified 15,854,827 dental visit records for 5,893,325 individuals, including their initial and following visits within 3 months. We excluded individuals with missing or inconsistent information on sex (*n* = 855), those who had died, emigrated, or had pre-existing GC before their first dental visit (*n* = 2050), those who had their first dental visit on the last day of the study period (*n* = 7), and those with other inflammations affecting the lips, tongue, or adjacent tissues (*n* = 2379). In total, 5291 (0.09%) subjects were excluded, leaving a final sample of 5,888,034 individuals for analysis. A detailed flowchart of the cohort assembly process is shown in Fig. 1. Participants were followed up through linkage of their data across multiple Swedish national registers using their unique identification numbers. Before being delivered to us, the linked datasets were de-identified, which cannot be traced back to personally identifiable information or other sensitive data. However, this linkage enabled us to track each subject from their first dental visit until the occurrence of the study outcome, death, emigration, or the end of the study period (December 31, 2016), whichever occurred first. The study was approved by the Ethical Review Board in Stockholm, Sweden (approval number: 2021–02491).

### Sibling-controlled analyses

In addition to the population comparison, we conducted a nested case-control study by linking incident GC cases to their full-siblings using the Swedish Multi-Generation Register (MGR), which includes parental data for children born since 1932 [18, 19]. In this design, we compared individuals diagnosed with GC during the follow-up period (cases) to their full-siblings who were alive and free of GC (controls), without a predetermined matching number. We focused on sibling pairs where one individual had GC and the other did not, regardless of their dental health status.

### Ascertainment of dental health condition and gastric cancer

Dental health diagnoses were extracted from the Dental Health Register (DHR) using specific diagnostic codes established by the Swedish Dental and Pharmaceutical Benefits Agency [17]. Participants were categorized hierarchically into the following groups: healthy; caries (diagnostic code: 4001, 4002, 4011, and 4012); root canal infection (diagnostic code: 3051); mild inflammation, which includes mucositis (diagnostic code: 3042), pericoronitis (diagnostic code: 3045), gingivitis (diagnostic code: 3041), stomatitis (diagnostic code: 3072 and 3073), and other inflammatory conditions of the gums and tissues around the teeth (diagnostic code: 3046); and periodontitis (diagnostic code: 3043 and 3044), based on the severity of damage and inflammation in the teeth and surrounding tissues [17, 20]. When multiple dental conditions were diagnosed, the most severe condition was prioritized as the baseline exposure, following this hierarchical order of severity (from least to most severe): healthy, dental caries, root canal infection, mild inflammation, and periodontitis. Among these dental health conditions, dental caries is not an inflammatory disease but rather a breakdown of the tooth enamel caused by bacterial metabolic activity. In subsequent interaction analysis, mild inflammation and periodontitis were categorized as “mild/severe inflammation,” while individuals with healthy teeth, caries, or root canal infection were categorized as “no inflammation/localized inflammation.” Baseline tooth counts were derived from the earliest visit, with longitudinal plausibility checks applied to resolve discrepancies with latter visits. Due to the severely skewed distribution of the tooth counts (i.e., most participants had few missing teeth, while a small subset had many), the number of remaining teeth at baseline was grouped into the following categories: > 27, > 24 and ≤ 27, > 20 and ≤ 24, > 14 and ≤ 20, and ≤ 14 teeth, in order to avoid overly sparse strata and ensuring robust statistical inference. Additionally, since the reason behind the missing number of teeth cannot be explained by available covariates, individuals without information on tooth number were categorized as “unknown.”

GC cases were identified from the Swedish Cancer Register (CR) using the International Classification of Diseases version 7 (ICD-7) code 151 (cardia: 1511, non-cardia: 151 except 1511) [21]. Clinical reporting requirements for clinicians in Sweden result in a very low underreporting rate of cancer cases (3.7%) in the CR [21].

### Covariates

Potential confounders for the association between dental health and GC include various socioeconomic characteristics of the participants. In our analyses, we considered covariates such as the participant’s baseline family income, categorized into age-specific tertiles (low, medium, and high), and education level (low: ≤ 9 years of primary education; medium: 10–11 years of education or high school education; high: ≥ 12 years, college education or higher; unknown). These data were obtained from the longitudinal integrated database for health insurance and labour market studies (LISA) [22]. Additionally, comorbidities, including alcohol-related disorders, smoking-related diseases, and chronic obstructive pulmonary disease, were extracted from the National Patient Register (NPR) [7, 23]. Family history of GC in the participants’ first-degree relatives was determined by linking to the MGR, CR, and the Swedish Total Population Register (TPR) [24]. Individuals with missing data on the variables were categorized as “unknown” to retain them in the analysis.

### Statistical analysis

Baseline characteristics of individuals in the DHR during the study period were summarized and presented by cancer status and sex. For cohort analyses, cox proportional hazards models using attained age as the timescale were applied to assess the association between dental health condition and the risk of GC, cardia GC and non-cardia GC, and hazard ratios (HRs) with 95% confidence intervals (CIs) were calculated. Schoenfeld residual plots did not show any violations of the proportional hazards assumption of the Cox models (Additional file 1: Fig. S1). For sibling-controlled analyses, conditional logistic regression models were utilized to estimate the odds ratios (ORs) and 95% CIs of GC in association with dental health status, conditioning on sibling groups. Both the cohort and sibling-controlled analyses were performed with adjustment for the aforementioned covariates. Individuals with healthy dental status or more than 27 remaining teeth served as the reference groups. The Cochran-Armitage test was employed to test for a linear trend in the risk of GC across increasingly severe dental health conditions, which ordered as an ordinal variable by clinical severity as healthy, caries, root canal infection, mild inflammation, and periodontitis.

Additionally, we evaluated the potential interaction effect between dental health condition and the number of remaining teeth on the risk of GC by comparing Cox regression models with and without a cross-product term using the analysis of variance test. We also assessed the risk of GC and its subtypes in relation to a combination of dental inflammatory conditions and the number of remaining teeth. In this analysis, individuals with healthy teeth, caries, or root canal infections were categorized as having "no inflammation/localized inflammation", while those with mild inflammation or periodontitis were classified as having "mild/severe inflammation". The number of remaining teeth was categorized as that in the main analyses.

In stratified analyses, we examined the associations of dental health condition and the number of remaining teeth with GC within different sex and age subgroups. In age-specific analyses, we stratified participants at 70 years to ensure sufficient GC case numbers in each exposure subgroup, a threshold further supported by its clinical relevance in geriatric health and disease risk assessment in prior research [25]. Additionally, to minimize selection bias, we conducted sensitivity analyses to assess the robustness of our main results by excluding incident cases diagnosed within 2 years after the first dental visit, or by excluding participants with missing covariate data.

Data management and statistical analyses were conducted using SAS (version 9.4), Stata 18 (basic edition), and R (version 4.3.1). A two-sided *p* value of less than 0.05 was considered statistically significant.

## Results

### Descriptive results

**Fig. 2:**
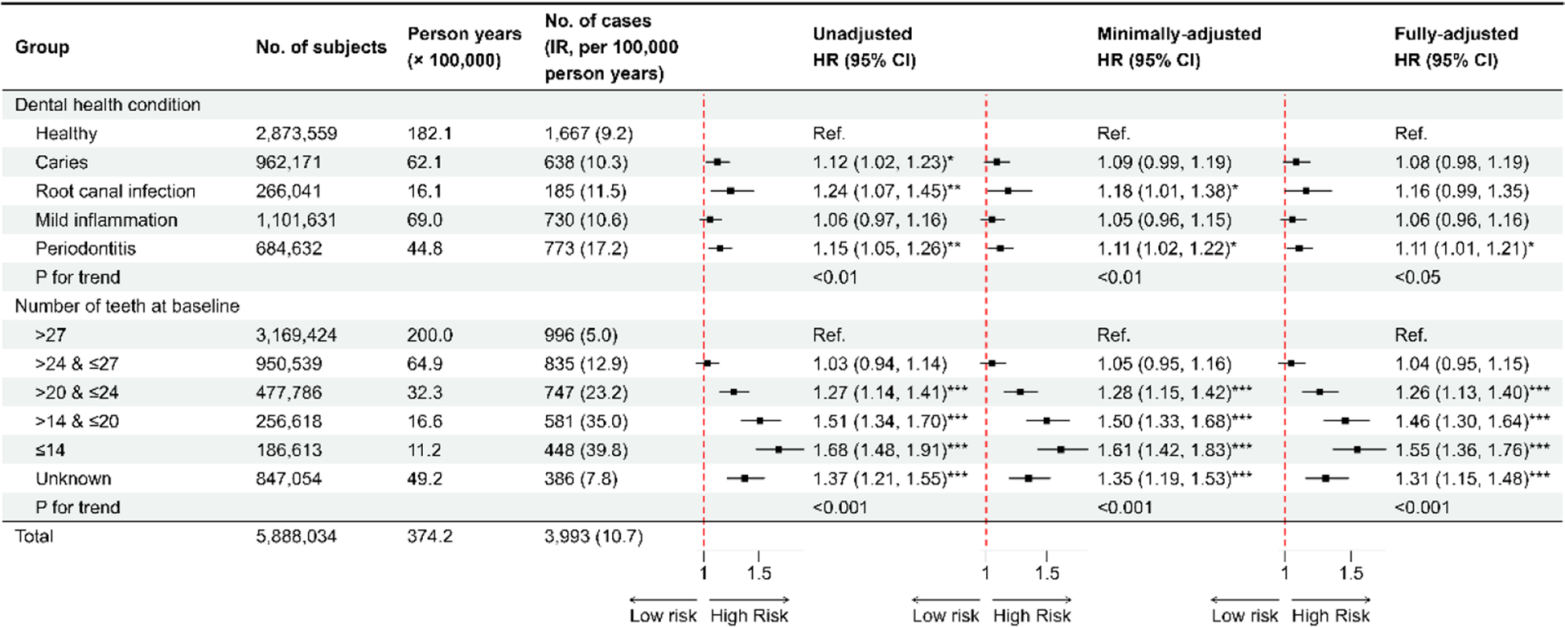
The association between baseline dental health condition and the risk of gastric cancer in the cohort analysis. All HR and 95% CI estimates were derived from Cox models with attained age as timescale: minimally adjusted models were adjusted for sex and age at entry; fully adjusted models were adjusted for sex, age at entry, family income, education, family history of gastric cancer, smoking-related diseases, and alcohol-related diseases. Trend analyses were performed by Cochran-Armitage test. The “unknown” group was excluded from the calculation of *p*-trend. Abbreviations: IR, incidence rate; HR, hazard ratio; CI, confidence interval. *: *p* < 0.05; **: *p* < 0.01; ***: *p* < 0.001

**Fig. 3:**
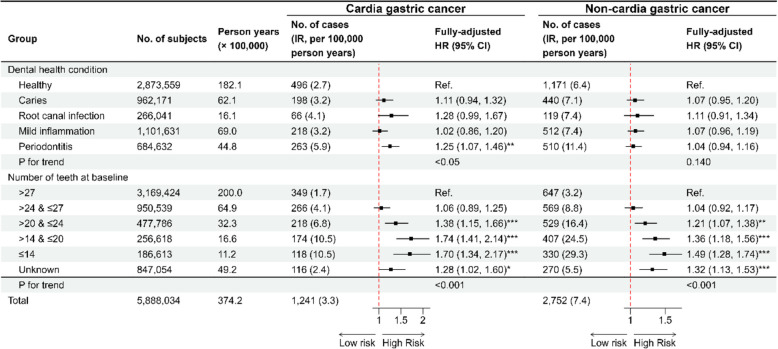
The association between baseline dental health condition and the risk of cardia and non-cardia gastric cancer in the cohort analysis. All HR and 95% CI estimates were derived from Cox models with attained age as timescale, adjusted for sex, age at entry, family income, education, family history of gastric cancer, smoking-related diseases, and alcohol-related diseases. Trend analyses were performed by Cochran-Armitage test. The “unknown” group was excluded from the calculation of *p*-trend. Abbreviations: IR, incidence rate; HR, hazard ratio; CI, confidence interval. *: *p* < 0.05; **: *p* < 0.01; ***: *p* < 0.001

The baseline characteristics of the participants described by cancer status and sex are presented in Table 1 and Additional file 1: Table S1. This prospective cohort included a total of 5,888,034 individuals, including 2,869,265 males and 3,018,769 females. The mean age of participants at baseline was 47.28 (± 18.87) years for those without GC, 65.88 (± 10.79) years for individuals with cardia GC, and 69.27 (± 11.78) years for those with non-cardia GC. The cohort accumulated a total of 37,419,745 person-years of follow-up, with an average follow-up duration of 6.36 (± 1.98) years. Among the participants, 962,171 individuals (16.3%) were diagnosed with dental caries, 266,041 individuals (4.5%) had root canal infections, 1,101,631 individuals (18.7%) presented with mild dental inflammation, and 684,632 individuals (11.6%) were diagnosed with periodontitis. The average number of remaining teeth at baseline was 27 (± 5) (847,054 missing, 14.4%). About half of the individuals (48.7%) had a high school education, with 2.8% missing (167,665). A total of 150,303 (2.6%) participants had alcohol-related diseases, 85,233 (1.4%) had smoking-related diseases, and 96,651 (1.6%) had a family history of GC (86,348 missing, 1.5%). During the follow-up period, we identified 3993 incident GC cases (incidence rate, IR: 10.7 per 100,000 person-years), including 1241 cardia GC cases (IR: 3.3 per 100,000 person-years) and 2752 non-cardia GC cases (IR: 7.4 per 100,000 person-years) (Figs. 2 and 3).

**Table 1:** Characteristics of individuals in the cohort identified from the Swedish Dental Health Register, 2009–2016

Characteristics	No gastric cancer (*N* = 5,884,041)	Cardia gastric cancer (*N* = 1241)	Non-cardia gastric cancer (*N* = 2752)	All participants (*N* = 5,888,034)
Follow-up years (mean ± SD)	6.36 (1.98)	3.87 (2.15)	3.62 (2.16)	6.36 (1.98)
Age (years, mean ± SD)	47.28 (18.87)	65.88 (10.79)	69.27 (11.78)	47.30 (18.87)
Dental health condition				
Healthy	2,871,892 (48.8)	496 (40.0)	1171 (42.6)	2,873,559 (48.8)
Caries	961,533 (16.3)	198 (16.0)	440 (16.0)	962,171 (16.3)
Root canal infection	265,856 (4.5)	66 (5.3)	119 (4.3)	266,041 (4.5)
Mild inflammation	1,100,901 (18.7)	218 (17.6)	512 (18.6)	1,101,631 (18.7)
Periodontitis	683,859 (11.6)	263 (21.2)	510 (18.5)	684,632 (11.6)
Number of teeth at baseline				
> 27	3,168,428 (53.8)	349 (28.1)	647 (23.5)	3,169,424 (53.8)
> 24 and ≤ 27	949,704 (16.1)	266 (21.4)	569 (20.7)	950,539 (16.1)
> 20 and ≤ 24	477,039 (8.1)	218 (17.6)	529 (19.2)	477,786 (8.1)
> 14 and ≤ 20	256,037 (4.4)	174 (14.0)	407 (14.8)	256,618 (4.4)
≤ 14	186,165 (3.2)	118 (9.5)	330 (12.0)	186,613 (3.2)
Unknown	846,668 (14.4)	116 (9.3)	270 (9.8)	847,054 (14.4)
Education				
Primary education and below (≤9 years)	1,049,156 (17.8)	390 (31.4)	1041 (37.8)	1,050,587 (17.8)
High school education (10 to 11 years)	2,866,837 (48.7)	496 (40.0)	1048 (38.1)	2,868,381 (48.7)
College education or higher (≥12 years)	1,800,628 (30.6)	251 (20.2)	522 (19.0)	1,801,401 (30.6)
Unknown	167,420 (2.8)	104 (8.4)	141 (5.1)	167,665 (2.8)
Family income				
Low	1,852,384 (31.5)	369 (29.7)	797 (29.0)	1,853,550 (31.5)
Medium	1,922,628 (32.7)	420 (33.8)	993 (36.1)	1,924,041 (32.7)
High	2,109,029 (35.8)	452 (36.4)	962 (35.0)	2,110,443 (35.8)
Family history of gastric cancer				
No	5,701,374 (96.9)	1146 (92.3)	2515 (91.4)	5,705,035 (96.9)
Yes	96,477 (1.6)	60 (4.8)	114 (4.1)	96,651 (1.6)
Unknown	86,190 (1.5)	35 (2.8)	123 (4.5)	86,348 (1.5)
Alcohol-related diseases				
No	5,733,870 (97.4)	1189 (95.8)	2672 (97.1)	5,737,731 (97.4)
Yes	150,171 (2.6)	52 (4.2)	80 (2.9)	150,303 (2.6)
Smoking-related diseases				
No	5,798,969 (98.6)	1179 (95.0)	2653 (96.4)	5,802,801 (98.6)
Yes	85,072 (1.4)	62 (5.0)	99 (3.6)	85,233 (1.4)

### Associations between dental health and GC and its subtypes

As shown in Figs. 2 and 3, our main analyses revealed a positive association between dental health condition and the risk of GC, and an inverse association between the number of remaining teeth and the risk of GC, although the effect size was slightly attenuated in the fully adjusted models.

In the fully adjusted models, associations between odontogenic inflammation and non-cardia GC were insignificant despite hazardous point estimate. The same pattern was observed for cardia GC except for periodontitis. Combining two subtypes did not change the direction or magnitude of the association. When compared to the healthy group, individuals suffering from periodontitis had an 11% (95% CI: 1% to 21%) and 25% (95% CI: 7% to 46%) increased risk of GC and cardia GC, respectively.

**Fig. 4:**
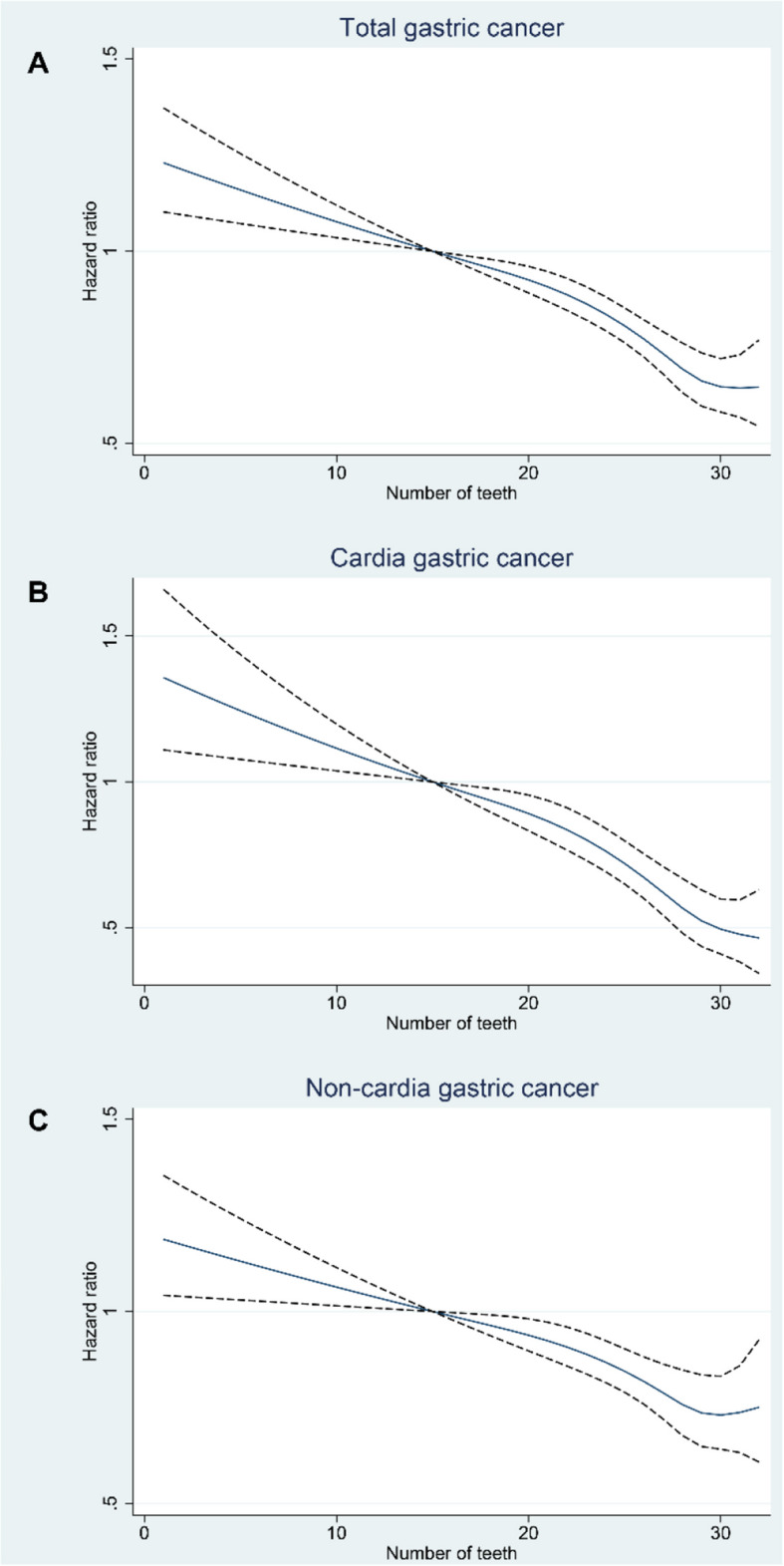
Exposure-response curve for the association of remaining tooth number at baseline with the risk of gastric cancer and its anatomical subtypes among individuals in the Swedish Dental Health Register, 2009–2016. **A** Total gastric cancer; **B** cardia gastric cancer; **C** non-cardia gastric cancer. The dark blue solid line represents the point estimates and the black dash lines indicate corresponding 95% CIs, which were derived from Cox models with attained age as timescale, adjusted for sex, age at entry, family income, education, family history of gastric cancer, smoking-related diseases, and alcohol-related diseases

The near-linear, negative exposure-response curves suggest decreasing trends in the risk of GC or its two subtypes as the number of remaining teeth at baseline increased (Fig. 4). When comparing individuals with more than 27 remaining teeth (the reference group), those with 24 to 27, 20 to 24, 14 to 20, and 14 or fewer remaining teeth had increased risks of GC by 4% (95% CI: − 5% to 15%), 26% (95% CI: 13% to 40%), 46% (95% CI: 30% to 64%), and 55% (95% CI: 36% to 76%), respectively. Similarly, the risks of cardia GC were increased by 6% (95% CI: − 11% to 25%), 38% (95% CI: 15% to 66%), 74% (95% CI: 41% to 114%), and 70% (95% CI: 34% to 117%) in these groups. For non-cardia GC, the risks were increased by 4% (95% CI: − 8% to 17%), 21% (95% CI: 7% to 38%), 36% (95% CI: 18% to 56%), and 49% (95% CI: 28% to 74%), respectively.

There was no significant interaction between dental inflammatory conditions and the number of remaining teeth on the risk of GC or its subtypes (*p* for interaction: 0.379 for total GC, 0.561 for cardia GC, and 0.345 for non-cardia GC). As shown in Additional file 1: Table S2, compared to individuals who had no inflammation and more than 27 remaining teeth, other groups generally showed increased risks of GC and its subtypes. For example, those with mild or severe dental inflammation and ≤ 14 remaining teeth had higher risks for total GC, cardia GC, and non-cardia GC, with HRs (95% CIs) of 1.68 (1.38, 2.03), 1.80 (1.25, 2.59), and 1.62 (1.29, 2.04), respectively.

### Sibling comparison

For the sibling-controlled study, we matched 4022 unaffected full-sibling controls for 1987 incident GC cases (Table 2). Consistent with the cohort analysis, individuals with dental health problem had a higher risk of developing GC. While some ORs were either insignificant or marginally significant due to limited statistical power, the overall trends were clear. For example, the ORs (95% CI) for GC were 1.25 (1.05, 1.49) for individuals with dental caries, compared to the healthy group. Additionally, we observed a gradual increase in GC risk with a decreasing number of teeth at baseline. Individuals with 20 to 24, 14 to 20, and ≤ 14 remaining teeth had increased risks of 28% (95% CI: 6% to 55%), 43% (95% CI: 13% to 81%), and 47% (95% CI: 10% to 95%) for GC compared to the reference group, which aligns with the estimates from the cohort analyses.

**Table 2:** The associations between dental health and gastric cancer in the sibling-controlled analysis

Group	No. of cases (%)	No. of sibling controls (%)	Unadjusted OR (95% CI)^a^	Fully adjusted OR (95% CI)^a,b^
Dental health condition				
Healthy	799 (40.2)	1725 (42.9)	Ref.	Ref.
Caries	329 (16.6)	561 (13.9)	1.29 (1.09, 1.52)**	1.25 (1.05, 1.49)*
Root canal infection	94 (4.7)	208 (5.2)	0.98 (0.75, 1.28)	0.99 (0.75, 1.30)
Mild inflammation	368 (18.5)	772 (19.2)	1.02 (0.87, 1.19)	0.98 (0.83, 1.15)
Periodontitis	397 (20.0)	756 (18.8)	1.18 (1.00, 1.38)*	1.12 (0.95, 1.32)
* p* for trend^c^			0.208	0.537
Number of teeth at baseline				
> 27	649 (32.7)	1419 (35.3)	Ref.	Ref.
> 24 and ≤ 27	474 (23.9)	1058 (26.3)	1.08 (0.93, 1.26)	1.03 (0.88, 1.22)
> 20 and ≤ 24	326 (16.4)	616 (15.3)	1.47 (1.22, 1.77)***	1.28 (1.06, 1.55)*
> 14 and ≤ 20	202 (10.2)	347 (8.6)	1.78 (1.42, 2.23)***	1.43 (1.13, 1.81)**
≤ 14	136 (6.8)	203 (5.0)	2.07 (1.58, 2.70)***	1.47 (1.10, 1.95)**
Unknown^d^	200 (10.1)	379 (9.4)	1.36 (1.11, 1.68)**	1.20 (0.96, 1.49)
* p* for trend^c^			<0.001	<0.05
Total	1987 (100.0)	4022 (100.0)		

*Abbreviations* *OR* odds ratio, *CI* confidence interval

^a^All OR and 95% CI estimates were derived from conditional logistic regression models

^b^Fully adjusted models were adjusted for sex, age at dental health assessment, family income, education, family history of gastric cancer, smoking-related diseases, and alcohol-related diseases

^c^Trend analyses were performed by Cochran-Armitage test

^d^The “unknown” group was excluded from the calculation of *p*-trend

*p < 0.05

**p < 0.01

***p < 0.001

### Subgroup analysis

The associations between dental health and GC, as well as its main subtypes, among different sex and age subgroups are shown in Additional file 1: Figs. S2–S5. Consistent with our main analyses, significant positive associations or trends were observed in most subgroups. For example, compared to the healthy group, the HRs (95% CI) for GC and cardia GC risk in the periodontitis group were 1.17 (1.05, 1.31) and 1.29 (1.08, 1.54) for male participants, and 1.25 (1.11, 1.41) and 1.44 (1.19, 1.74) for individuals under 70 years old. Additionally, compared to participants with more than 27 remaining teeth, those with ≤ 14 remaining teeth at baseline had HRs (95% CI) of 1.60 (1.36, 1.88), 1.58 (1.20, 2.09), and 1.59 (1.30, 1.94) for GC, cardia GC, and non-cardia GC, respectively, in male participants, and 1.75 (1.41, 2.17), 1.90 (1.35, 2.69), and 1.67 (1.27, 2.19) in participants under 70 years old. However, the associations between dental health condition and non-cardia GC in males, females, and those over 70 years old, as well as the associations between dental health condition and total GC in females and those over 70, were only marginally significant.

### Sensitivity analysis

Our sensitivity analysis, which excluded incident cases and follow-up data from the first 2 years (Additional file 1: Fig. S6) as well as participants with missing covariate data (Additional file 1: Fig. S7), further supported the significant associations between poor dental health, fewer remaining teeth at baseline, and increased risks of GC and its subtypes. Despite reduced stability in some association estimates, all these findings were consistent with our primary results.

## Discussion

This nationwide prospective cohort and sibling-controlled nested case-control study investigated the association between dental health and GC in the Swedish adult population, focusing on both overall GC and its anatomical subtypes. Our findings suggest that individuals with odontogenic inflammation are at higher risk of developing GC, particularly the cardia type. Additionally, we observed a gradual increase in the risk of both cardia and non-cardia GC as the number of remaining teeth decreases. These associations were consistently observed in sibling comparisons, across various sex and age subgroups, and in sensitivity analyses. To our knowledge, this is the largest population-based prospective cohort study and the first to use sibling-controlled analysis to investigate this association, providing significant evidence on the risk factors for gastric carcinogenesis.

The prevalence of dental caries and periodontitis in the study population was relatively low compared to global estimates but aligned with findings from other Swedish registry-based studies [26, 27]. This discrepancy may be attributed to the inherent limitations of the DHR, which excludes individuals with limited access to dental care. Furthermore, the DHR’s diagnostic criteria for periodontitis prioritize advanced cases, potentially leading to an underestimation of mild or early-stage disease prevalence. Additionally, Sweden’s high rate of dental care utilization and widespread implementation of preventive measures likely contribute to the lower disease prevalence observed compared to the global average.

Previous studies have indicated that poor dental health may contribute to the development of GC. For example, several epidemiologic studies have reported positive associations between periodontitis, tooth loss, dental caries, denture-associated lesions, or poor oral hygiene and the risk of gastric precancerous lesions and GC [28–30]. A nested case-control study in a Korean population found that participants with a history of chronic periodontitis were more likely to develop GC [31]. Additionally, another prospective cohort study demonstrated a significant association between tooth loss and an elevated risk of GC, with tooth loss linked to a 30% increase in the risk of cardia GC and an 80% increase in the risk of non-cardia GC [14]. Our findings align with these studies in demonstrating that odontogenic inflammations are associated with an increased risk of GC, and further reveal a dose-response relationship between the number of remaining teeth and the risk of GC.

While prior research usually focused on tooth count as a simple marker of dental health in relation to GC, these studies have largely overlooked the distinct etiological differences between cardia and non-cardia GC subtypes and have rarely accounted for unmeasured confounding factors, such as familial or genetic influences [15, 32]. Notably, to our best knowledge, no prior work has reported subtype-specific associations between dental health indicators and GC risk. Our study addresses this critical issue by demonstrating that lower tooth count is associated with elevated GC risk for both cardia and non-cardia subtypes, while periodontitis exhibits a stronger link to cardia cancer, thereby challenging the assumption of uniform dental health associations across GC subtypes. By employing both cohort design and sibling-controlled analyses—which account for shared familial factors (e.g., genetics, early-life environment)—our findings strengthen causal inference compared to traditional observational studies. Importantly, this study identifies dental health as a potentially modifiable risk factor for GC, distinct from non-modifiable factors like age or genetics, suggesting that interventions targeting oral hygiene (e.g., periodontal treatment, dental care promotion) could be integrated into preventive strategies, particularly in high-risk populations.

The observed site-specific association between periodontitis and cardia GC, but not non-cardia GC, supports hypotheses regarding anatomical and etiological differences underlying these subtypes. The proximity of the cardia to the oral cavity may facilitate direct exposure to periodontal pathogens or inflammatory mediators, as well as influence by gastroesophageal reflux, whereas non-cardia gastric tumors are more strongly linked to *Helicobacter pylori* infection and subsequent gastric mucosal atrophy [33]. In addition, while most of our estimates suggested elevated risks across subtypes, the limited number of non-cardia cases in certain exposure categories likely reduced statistical power, resulting in wider confidence intervals and marginally significant estimates. Furthermore, we observed that root canal infection is associated with higher risk of GC than periodontitis. While we cannot rule out biological plausibility (e.g., chronic endodontic infections may introduce distinct oral-gut microbial or inflammatory pathways), the limited sample size precludes definitive conclusions. While this is the largest cohort study on this topic, future pooled analyses or extended follow-up are needed to enhance statistical power for subgroup analyses and validate findings in underrepresented populations.

Emerging epidemiologic and laboratory studies suggest that the underlying mechanisms for the association between dental health condition and gastrointestinal cancers are likely intricate and multifaceted, involving alterations in the oral and gut microbiomes, chronic inflammation, and dietary changes due to tooth loss [31, 34, 35]. First, patients with poor dental health often experience changes in microbial diversity of dental plaque and saliva, along with a greater burden of periodontal pathogens, which may promote gastric carcinogenesis [36]. Second, congenital tooth agenesis has been previously linked to an increased risk of certain cancers, which could potentially explain some of the observed associations between missing teeth and GC risk in our study [37]. Third, long-standing inflammation associated with dental health condition has been proposed as a risk or prerequisite factor in driving GC development, growth, and progression [38, 39]. Elevated secretion of inflammatory markers, such as tumor necrosis factor-α and interleukins-1α and -1β, have been observed in GC patients [40, 41]. Moreover, dental health issues, such as caries and periodontal disease, can lead to tooth loss, which might impair chewing ability and alter dietary habits. Insufficient chewing might lead to swallowing large pieces of food, which can irritate or damage the gastrointestinal tract mucosa, ultimately increasing the risk of GC [14, 32, 42]. Thus, it is biologically plausible that poor dental health could be a significant contributor to the development of GC.

The strengths of our study include its status as the largest prospective cohort study to date on this topic, which provides stronger evidence for a causal relationship. Moreover, the use of sibling controls in the nested case-control analysis effectively mitigates the potential impact of familial confounders and other unmeasured factors shared by siblings. Additionally, dental health information was collected from the Swedish DHR, a comprehensive nationwide database that includes clinical records for nearly all individuals who have visited a dentist in Sweden [17]. The CR, which has been widely used in population-based research on cancer incidence and survival, is recognized for its high reliability and completeness in previous studies, with approximately 99% of cases verified and minimal underreporting [21]. Both the DHR and CR utilize ICD codes for disease diagnoses, ensuring greater accuracy and reliability compared to self-reported data.

However, several limitations should be acknowledged in our study. First, our data were obtained from multiple Swedish nationwide registers, which did not include information on some important lifestyle factors, such as diet, alcohol consumption, and smoking. This limitation prevented us from directly adjusting for these factors. We used proxies for alcohol and smoking in our analyses to address this issue as much as possible. Further research with more detailed information on these potential confounders is needed. Second, the development of cancer can take many years, even more than a decade. Although our study had a mean follow-up time of 6.36 years, it may not have been long enough to identify long-term GC risk associated with poor oral health. However, the minimal changes in effect estimates in our sensitivity analysis after excluding GC cases diagnosed within 2 years suggest that reverse causality is unlikely to explain our findings. Third, our study did not include individuals with limited access to dental care (e.g., non-attenders due to socioeconomic barriers or rural residency), which could underestimate true population-level prevalence. However, previous studies have reported high coverage rates for the Dental Health Register used in our study [17], suggesting that this issue is unlikely to substantially affect the accuracy of our estimates. Fourth, this study could not account for the impact of dental treatments, such as periodontal therapy, fillings, and preventive care, which may modify inflammatory burden and confound associations. Additionally, although our effect estimates were weighted by follow-up time of the individuals, we were not able to directly assess the relationship between the duration of dental inflammation and GC risk, as the exact onset time of these chronic and largely irreversible conditions could not be retrospectively determined.

## Conclusions

This population-based cohort study, supplemented with sibling-controlled analyses, provides novel evidence that lower tooth count is associated with an increased risk of GC, with similar associations observed for both cardia and non-cardia subtypes. Additionally, among the odontogenic diseases evaluated, periodontitis emerged as a stable risk factor for gastric cancer, particularly for the cardia subtype. Further studies are needed to investigate the underlying biological mechanisms for these associations.

## Supplementary Information

Additional file 1: Tables S1 and S2 and Figures S1–S7. Table S1 Baseline characteristics of individuals stratified by sex. Table S2 Interaction analysis. Fig. S1 Schoenfeld residual plots. Fig. S2 Subgroup analysis (male). Fig. S3 Subgroup analysis (female). Fig. S4 Subgroup analysis (aged ≤ 70 years). Fig. S5 Subgroup analysis (aged > 70 years). Fig. S6 Sensitivity analysis by excluding incident cases and follow-up data observed within the first 2 years. Fig. S7 Sensitivity analysis by excluding participants with missing covariate data.

## Data Availability

Data are from the Swedish population and health registers. According to the Swedish law, data cannot be put into a public data repository but are available by applying through Statistics Sweden or the Swedish National Board of Health and Welfare.

## Abbreviations

GC: Gastric cancer
MGR: Multi-Generation Register
DHR: Dental Health Register
CR: Cancer Register
ICD: International Classification of Diseases
LISA: Longitudinal integrated database for health insurance and labour market studies
NPR: National Patient Register
TPR: Total Population Register
HR: Hazard ratio
OR: Odds ratio
CI: Confidence interval
