# Supplementary material for: The association between poor dental health and gastric cancer risk: a nationwide cohort and sibling-controlled study
Source: BMC Med. 2025 Jul 21;23:434. doi: 10.1186/s12916-025-04273-x (PMC12282018; doi:10.1186/s12916-025-04273-x)
Supplement: Supplementary file 1 — Additional file 1: Tables S1 and S2 and Figures S1–S7. Table S1 Baseline characteristics of individuals stratified by sex. Table S2 Interaction analysis. Fig. S1 Schoenfeld residual plots. Fig. S2 Subgroup analysis (male). Fig. S3 Subgroup analysis (female). Fig. S4 Subgroup analysis (aged ≤ 70 years). Fig. S5 Subgroup analysis (aged > 70 years). Fig. S6 Sensitivity analysis by excluding incident cases and follow-up data observed within the first 2 years. Fig. S7 Sensitivity analysis by excluding participants with missing covariate data. [file 12916_2025_4273_MOESM1_ESM.pdf]

Supplementary materials

The association between poor dental health and gastric cancer risk: a nationwide cohort and sibling-controlled study

Zengliang Ruan [1,2], Jianfeng Xie [2,3], Jingru Yu [2], Li Yin [2], Dariush Nasrollahzadeh Nesheli [2], Weimin Ye [2,4,*]

[1] Key Laboratory of Environmental Medicine and Engineering of Ministry of Education, and Department of Epidemiology and Health Statistics, School of Public Health, Southeast University, Nanjing, Jiangsu Province, China

[2] Department of Medical Epidemiology and Biostatistics, Karolinska Institutet, Stockholm, Sweden

[3] AIDS/STD Prevention and Treatment Institute, Fujian Provincial Center for Disease Control and Prevention, Fuzhou, China

[4] Department of Epidemiology and Health Statistics, School of Public Health, Fujian Medical University, Fuzhou, China

* **Corresponding author:** Weimin Ye, Department of Medical Epidemiology and Biostatistics, Karolinska Institutet, Nobels väg 12a, 17165 Solna, Stockholm, Sweden. Email: weimin.ye@ki.se

Table of Contents

Table S1. Baseline characteristics of individuals stratified by sex.

Table S2. The risk of gastric cancer and its subtypes in relation to a combination of dental inflammatory conditions and number of remaining teeth.

Fig. S1. Schoenfeld residual plots.

Fig. S2. The association between baseline dental health condition and the risk of gastric cancer among male individuals in the Swedish Dental Health Register, 2009-2016.

Fig. S3. The association between baseline dental health condition and the risk of gastric cancer among female individuals in the Swedish Dental Health Register, 2009-2016.

Fig. S4. The association between baseline dental health condition and the risk of gastric cancer among individuals aged ≤70 years old in the Swedish Dental Health Register, 2009-2016.

Fig. S5. The association between baseline dental health condition and the risk of gastric cancer among individuals aged >70 years old in the Swedish Dental Health Register, 2009-2016.

Fig. S6. The association between baseline dental health condition and the risk of gastric cancer after excluding incident cases and follow-up data observed within the first two years.

Fig. S7. The association between baseline dental health condition and the risk of gastric cancer after excluding participants with missing covariate data.

Table S1. Baseline characteristics of individuals stratified by sex.

Characteristics	Male (N=2,869,265)	Female (N=3,018,769)	All participants (N=5,888,034)
**Follow-up years (mean ± SD)**	6.27 (2.03)	6.44 (1.92)	6.36 (1.98)
**Age (years, mean ± SD)**	46.79 (18.48)	47.78 (19.22)	47.30 (18.87)
**Dental health condition**			
Healthy	1,331,757 (46.4)	1,541,802 (51.1)	2,873,559 (48.8)
Caries	485,794 (16.9)	476,377 (15.8)	962,171 (16.3)
Root canal infection	146,805 (5.1)	119,236 (3.9)	266,041 (4.5)
Mild inflammation	551,144 (19.2)	550,487 (18.2)	1,101,631 (18.7)
Periodontitis	353,765 (12.3)	330,867 (11.0)	684,632 (11.6)
**Number of teeth at baseline**			
>27	1,610,280 (56.1)	1,559,144 (51.6)	3,169,424 (53.8)
>24 &≤27	427,652 (14.9)	522,887 (17.3)	950,539 (16.1)
>20 &≤24	206,937 (7.2)	270,849 (9.0)	477,786 (8.1)
>14 &≤20	112,178 (3.9)	144,440 (4.8)	256,618 (4.4)
≤14	85,380 (3.0)	101,233 (3.4)	186,613 (3.2)
Unknown	426,838 (14.9)	420,216 (13.9)	847,054 (14.4)
**Education**			
Primary education and below (≤9 years)	545,321 (19.0)	505,266 (16.7)	1,050,587 (17.8)
High school education (10 to 11 years)	1,443,494 (50.3)	1,424,887 (47.2)	2,868,381 (48.7)
College education or higher (≥ 12 years)	797,579 (27.8)	1,003,822 (33.3)	1,801,401 (30.6)
Unknown	82,871 (2.9)	84,794 (2.8)	167,665 (2.8)
**Family income**			
Low	800,553 (27.9)	1,052,997 (34.9)	1,853,550 (31.5)
Medium	971,484 (33.9)	952,557 (31.6)	1,924,041 (32.7)
High	1,097,228 (38.2)	1,013,215 (33.6)	2,110,443 (35.8)
**Family history of gastric cancer**			
No	2,781,899 (97.0)	2,923,136 (96.8)	5,705,035 (96.9)
Yes	47,229 (1.6)	49,422 (1.6)	96,651 (1.6)
Unknown	40,137 (1.4)	46,211 (1.5)	86,348 (1.5)
**Alcohol-related diseases**			
No	2,776,163 (96.8)	2,961,568 (98.1)	5,737,731 (97.4)
Yes	93,102 (3.2)	57,201 (1.9)	150,303 (2.6)
**Smoking-related diseases**			
No	2,828,669 (98.6)	2,974,132 (98.5)	5,802,801 (98.6)
Yes	40,596 (1.4)	44,637 (1.5)	85,233 (1.4)

Table S2. The risk of gastric cancer and its subtypes in relation to a combination of dental inflammatory conditions and number of remaining teeth [a].

Group [b]	Total gastric cancer		Cardia gastric cancer		Non-cardia gastric cancer	
	No. of cases	HR (95% CI) [c]	No. of cases	HR (95% CI) [c]	No. of cases	HR (95% CI) [c]
No inflammation/Localized inflammation & >27	616	Ref.	212	Ref.	404	Ref.
No inflammation/Localized inflammation & 25-27	510	1.08 (0.95, 1.22)	148	1.00 (0.80, 1.25)	362	1.11 (0.96, 1.29)
No inflammation/Localized inflammation & 21-24	431	1.25 (1.09, 1.42)**	128	1.42 (1.12, 1.79)**	303	1.18 (1.00, 1.39)*
No inflammation/Localized inflammation 15-20	365	1.53 (1.32, 1.76)***	112	1.87 (1.44, 2.42)***	253	1.40 (1.18, 1.67)***
No inflammation/Localized inflammation & ≤14	296	1.58 (1.35, 1.85)***	79	1.78 (1.33, 2.38)***	217	1.50 (1.25, 1.81)***
No inflammation/Localized inflammation & Unknown	272	1.39 (1.19, 1.61)***	81	1.36 (1.04, 1.78)*	191	1.40 (1.17, 1.67)***
Mild/Severe inflammation & >27	380	1.11 (0.97, 1.26)	137	1.12 (0.90, 1.40)	243	1.10 (0.93, 1.29)

Mild/Severe inflammation & 25-27	325	1.10 (0.95, 1.27)	118	1.27 (1.00, 1.61)*	207	1.03 (0.86, 1.22)
Mild/Severe inflammation & 21-24	316	1.41 (1.22, 1.64)***	90	1.49 (1.14, 1.94)**	226	1.38 (1.16, 1.65)***
Mild/Severe inflammation 15-20	216	1.50 (1.27, 1.77)***	62	1.74 (1.29, 2.36)***	154	1.40 (1.15, 1.72)***
Mild/Severe inflammation & ≤14	152	1.68 (1.38, 2.03)***	39	1.80 (1.25, 2.59)**	113	1.62 (1.29, 2.04)***
Mild/Severe inflammation & Unknown	114	1.29 (1.04, 1.59)*	35	1.28 (0.88, 1.88)	79	1.29 (1.00, 1.66)
Total	3,993		1,241		2,752	

[a]: *P* for interaction was 0.379 for total gastric cancer, 0.561 for cardia gastric cancer, and 0.345 for non-cardia gastric cancer.

[b]: Mild/Severe inflammation encompasses both mild inflammation and periodontitis; no inflammation/localized inflammation includes healthy, caries, and root canal infection.

[c]: All HR and 95% CI estimates were derived from Cox models with attained age as time-scale, and adjusted for sex, age at entry, family income, education, family history of gastric cancer, smoking-related diseases, and alcohol-related diseases.

*: $p < 0.05$; **: $p < 0.01$; ***: $p < 0.001$.

Abbreviations: HR, hazard ratio; CI, confidence interval.

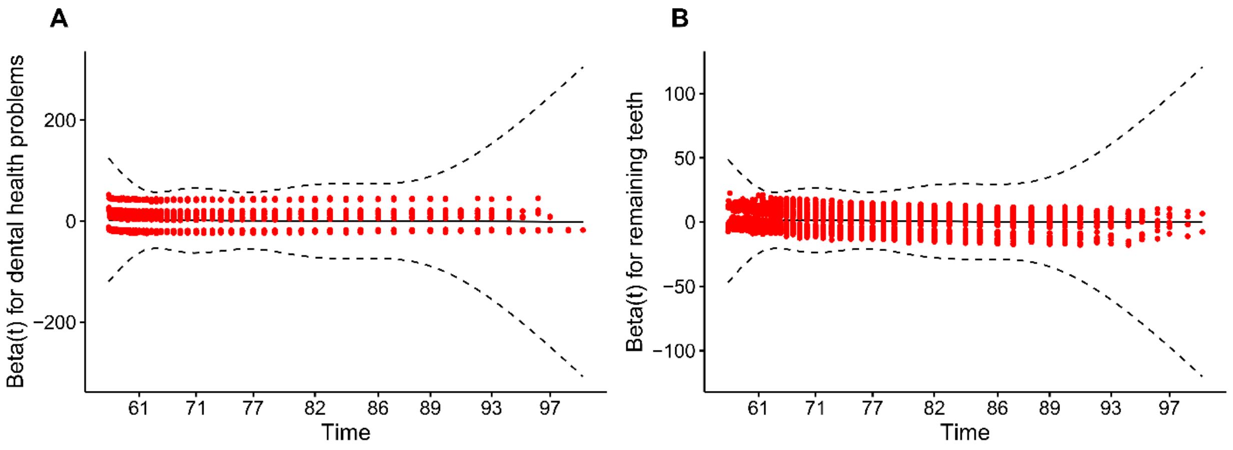

Fig. S1. Schoenfeld residual plots. (A) Dental health condition; (B) Remaining teeth.

Group	No. of subjects	Person years (× 100,000)	All gastric cancer: No. of cases (IR, per 100,000 person years)	All gastric cancer: Fully-adjusted HR (95% CI)	Cardia gastric cancer: No. of cases (IR, per 100,000 person years)	Cardia gastric cancer: Fully-adjusted HR (95% CI)	Non-cardia gastric cancer: No. of cases (IR, per 100,000 person years)	Non-cardia gastric cancer: Fully-adjusted HR (95% CI)
Dental health condition								
Healthy	1,331,757	83.5	953 (11.4)	Ref.	357 (4.3)	Ref.	596 (7.1)	Ref.
Caries	485,794	30.9	393 (12.7)	1.07 (0.95, 1.21)	150 (4.8)	1.11 (0.91, 1.35)	243 (7.9)	1.04 (0.89, 1.22)
Root canal infection	146,805	8.6	125 (14.5)	1.20 (1.00, 1.45)	45 (5.2)	1.12 (0.82, 1.53)	80 (9.3)	1.26 (0.99, 1.59)
Mild inflammation	551,144	34.0	444 (13.0)	1.08 (0.96, 1.21)	168 (4.9)	1.05 (0.87, 1.27)	276 (8.1)	1.10 (0.95, 1.27)
Periodontitis	353,765	22.7	512 (22.5)	1.17 (1.05, 1.31)**	208 (9.2)	1.29 (1.08, 1.54)**	304 (13.4)	1.10 (0.95, 1.27)
P for trend				<0.01		<0.01		0.787
Number of teeth at baseline								
>27	1,610,280	100.6	650 (6.5)	Ref.	279 (2.8)	Ref.	371 (3.7)	Ref.
>24 & ≤27	427,652	28.9	484 (16.7)	0.98 (0.86, 1.11)	192 (6.6)	0.99 (0.81, 1.20)	292 (10.1)	0.97 (0.83, 1.14)
>20 & ≤24	206,937	13.8	450 (32.7)	1.25 (1.09, 1.43)**	165 (12.0)	1.36 (1.10, 1.68)**	285 (20.7)	1.19 (1.00, 1.41)*
>14 & ≤20	112,178	7.1	326 (45.9)	1.36 (1.17, 1.58)***	119 (16.8)	1.52 (1.19, 1.95)***	207 (29.2)	1.27 (1.05, 1.54)*
≤14	85,380	5.0	286 (57.0)	1.60 (1.36, 1.88)***	86 (17.1)	1.58 (1.20, 2.09)**	200 (39.9)	1.59 (1.30, 1.94)***
Unknown	426,838	24.4	231 (9.5)	1.23 (1.05, 1.44)*	87 (3.6)	1.23 (0.95, 1.59)	144 (5.9)	1.22 (1.00, 1.50)
P for trend				<0.001		<0.001		0.696
Total	2,869,265	179.8	2,427 (13.5)		928 (5.2)		1,499 (8.3)	

Fig. S2. The association between baseline dental health condition and the risk of gastric cancer among male individuals in the Swedish Dental Health Register, 2009-2016. All HR and 95% CI estimates were derived from Cox models with attained age as time-scale, adjusted for sex, age at entry, family income, education, family history of gastric cancer, smoking-related diseases, and alcohol-related diseases. Trend analyses were performed by Cochran-Armitage test. The 'unknown' group was excluded from the calculation of *p*-trend. Abbreviations: IR, incidence rate; HR, hazard ratio; CI, confidence interval. *: $p < 0.05$; **: $p < 0.01$; ***: $p < 0.001$.

Group	No. of subjects	Person years (× 100,000)	All gastric cancer: No. of cases (IR, per 100,000 person years)	All gastric cancer: Fully-adjusted HR (95% CI)	Cardia gastric cancer: No. of cases (IR, per 100,000 person years)	Cardia gastric cancer: Fully-adjusted HR (95% CI)	Non-cardia gastric cancer: No. of cases (IR, per 100,000 person years)	Non-cardia gastric cancer: Fully-adjusted HR (95% CI)
Dental health condition								
Healthy	1,541,802	98.7	714 (7.2)	Ref.	139 (1.4)	Ref.	575 (5.8)	Ref.
Caries	476,377	31.1	245 (7.9)	1.11 (0.95, 1.29)	48 (1.5)	1.13 (0.80, 1.59)	197 (6.3)	1.10 (0.93, 1.31)
Root canal infection	119,236	7.5	60 (8.0)	1.09 (0.83, 1.42)	21 (2.8)	1.90 (1.18, 3.04)**	39 (5.2)	0.89 (0.64, 1.23)
Mild inflammation	550,487	35.0	286 (8.2)	1.03 (0.89, 1.19)	50 (1.4)	0.93 (0.66, 1.30)	236 (6.7)	1.05 (0.90, 1.23)
Periodontitis	330,867	22.1	261 (11.8)	1.00 (0.87, 1.16)	55 (2.5)	1.13 (0.82, 1.56)	206 (9.3)	0.97 (0.83, 1.15)
P for trend				0.772		0.780		0.850
Number of teeth at baseline								
>27	1,559,144	99.4	346 (3.5)	Ref.	70 (0.7)	Ref.	276 (2.8)	Ref.
>24 & ≤27	522,887	36.0	351 (9.7)	1.15 (0.98, 1.35)	74 (2.1)	1.32 (0.93, 1.87)	277 (7.7)	1.11 (0.93, 1.33)
>20 & ≤24	270,849	18.5	297 (16.0)	1.28 (1.07, 1.52)**	53 (2.9)	1.49 (1.01, 2.21)*	244 (13.2)	1.23 (1.01, 1.49)*
>14 & ≤20	144,440	9.5	255 (26.9)	1.62 (1.34, 1.95)***	55 (5.8)	2.53 (1.68, 3.82)***	200 (21.1)	1.45 (1.17, 1.79)***
≤14	101,233	6.2	162 (26.0)	1.46 (1.18, 1.82)***	32 (5.1)	2.19 (1.35, 3.57)**	130 (20.9)	1.32 (1.04, 1.69)*
Unknown	420,216	24.8	155 (6.3)	1.44 (1.18, 1.76)***	29 (1.2)	1.46 (0.91, 2.33)	126 (5.1)	1.42 (1.14, 1.78)**
P for trend				<0.001		<0.001		<0.001
Total	3,018,769	194.4	1,566 (8.1)		313 (1.6)		1,253 (6.4)	

Fig. S3. The association between baseline dental health condition and the risk of gastric cancer among female individuals in the Swedish Dental Health Register, 2009-2016. All HR and 95% CI estimates were derived from Cox models with attained age as time-scale, adjusted for sex, age at entry, family income, education, family history of gastric cancer, smoking-related diseases, and alcohol-related diseases. Trend analyses were performed by Cochran-Armitage test. The 'unknown' group was excluded from the calculation of *p*-trend. Abbreviations: IR, incidence rate; HR, hazard ratio; CI, confidence interval. *: p < 0.05; **: p < 0.01; ***: p < 0.001.

Group	No. of subjects	Person years (× 100,000)	All gastric cancer: No. of cases (IR, per 100,000 person years)	All gastric cancer: Fully-adjusted HR (95% CI)	Cardia gastric cancer: No. of cases (IR, per 100,000 person years)	Cardia gastric cancer: Fully-adjusted HR (95% CI)	Non-cardia gastric cancer: No. of cases (IR, per 100,000 person years)	Non-cardia gastric cancer: Fully-adjusted HR (95% CI)
Dental health condition								
Healthy	2,521,324	160.5	847 (5.3)	Ref.	299 (1.9)	Ref.	548 (3.4)	Ref.
Caries	841,867	54.7	347 (6.3)	1.18 (1.04, 1.34)*	132 (2.4)	1.23 (1.00, 1.52)	215 (3.9)	1.15 (0.98, 1.35)
Root canal infection	236,265	14.3	108 (7.5)	1.20 (0.98, 1.47)	46 (3.2)	1.33 (0.96, 1.82)	62 (4.3)	1.12 (0.86, 1.46)
Mild inflammation	968,015	60.5	415 (6.9)	1.16 (1.03, 1.31)*	153 (2.5)	1.16 (0.94, 1.42)	262 (4.3)	1.17 (1.00, 1.36)*
Periodontitis	542,229	36.0	449 (12.5)	1.25 (1.11, 1.41)***	188 (5.2)	1.44 (1.19, 1.74)***	261 (7.3)	1.14 (0.98, 1.33)
P for trend				<0.001		<0.001		0.073
Number of teeth at baseline								
>27	3,070,284	193.7	790 (4.1)	Ref.	299 (1.5)	Ref.	491 (2.5)	Ref.
>24 & ≤27	801,952	55.1	534 (9.7)	1.09 (0.97, 1.23)	203 (3.7)	1.13 (0.94, 1.37)	331 (6.0)	1.07 (0.92, 1.24)
>20 & ≤24	300,802	20.8	336 (16.1)	1.41 (1.22, 1.61)***	124 (6.0)	1.50 (1.20, 1.87)***	212 (10.2)	1.35 (1.14, 1.61)***
>14 & ≤20	103,577	7.1	186 (26.0)	1.75 (1.47, 2.08)***	79 (11.1)	2.07 (1.57, 2.71)***	107 (15.0)	1.58 (1.26, 1.97)***
≤14	63,718	4.2	112 (26.6)	1.75 (1.41, 2.17)***	41 (9.7)	1.90 (1.35, 2.69)***	71 (16.8)	1.67 (1.27, 2.19)***
Unknown	769,367	45.1	208 (4.6)	1.26 (1.07, 1.48)**	72 (1.6)	1.17 (0.89, 1.53)	136 (3.0)	1.31 (1.07, 1.60)**
P for trend				<0.001		<0.001		<0.001
Total	5,109,700	326.0	2,166 (6.6)		818 (2.5)		1,348 (4.1)	

Forest plot axes: All gastric cancer (1, 1.5, 2); Cardia gastric cancer (1, 1.5, 2, 2.5); Non-cardia gastric cancer (1, 1.5, 2). ← Low risk | High Risk →

Fig. S4. The association between baseline dental health condition and the risk of gastric cancer among individuals aged ≤70 years old in the Swedish Dental Health Register, 2009-2016. All HR and 95% CI estimates were derived from Cox models with attained age as time-scale, adjusted for sex, age at entry, family income, education, family history of gastric cancer, smoking-related diseases, and alcohol-related diseases. Trend analyses were performed by Cochran-Armitage test. The 'unknown' group was excluded from the calculation of *p*-trend. Abbreviations: IR, incidence rate; HR, hazard ratio; CI, confidence interval. *: $p < 0.05$; **: $p < 0.01$; ***: $p < 0.001$.

Group	No. of subjects	Person years (× 100,000)	All gastric cancer: No. of cases (IR, per 100,000 person years)	All gastric cancer: Fully-adjusted HR (95% CI)	Cardia gastric cancer: No. of cases (IR, per 100,000 person years)	Cardia gastric cancer: Fully-adjusted HR (95% CI)	Non-cardia gastric cancer: No. of cases (IR, per 100,000 person years)	Non-cardia gastric cancer: Fully-adjusted HR (95% CI)
Dental health condition								
Healthy	352,235	21.6	820 (37.9)	Ref.	197 (9.1)	Ref.	623 (28.8)	Ref.
Caries	120,304	7.4	291 (39.3)	0.98 (0.85, 1.13)	66 (8.9)	0.93 (0.69, 1.24)	225 (30.4)	1.00 (0.85, 1.17)
Root canal infection	29,776	1.8	77 (43.2)	1.14 (0.90, 1.45)	20 (11.2)	1.23 (0.78, 1.96)	57 (32.0)	1.11 (0.84, 1.46)
Mild inflammation	133,616	8.5	315 (37.0)	0.95 (0.82, 1.09)	65 (7.6)	0.78 (0.58, 1.06)	250 (29.4)	1.00 (0.85, 1.17)
Periodontitis	142,403	8.9	324 (36.5)	0.96 (0.84, 1.10)	75 (8.4)	0.95 (0.72, 1.24)	249 (28.0)	0.96 (0.83, 1.12)
P for trend				0.955		0.498		0.665
Number of teeth at baseline								
>27	99,140	6.3	206 (32.6)	Ref.	50 (7.9)	Ref.	156 (24.7)	Ref.
>24 & ≤27	148,587	9.9	301 (30.5)	0.91 (0.76, 1.09)	63 (6.4)	0.77 (0.52, 1.13)	238 (24.1)	0.96 (0.78, 1.18)
>20 & ≤24	176,984	11.5	411 (35.9)	1.04 (0.87, 1.23)	94 (8.2)	1.06 (0.74, 1.51)	317 (27.7)	1.03 (0.84, 1.26)
>14 & ≤20	153,041	9.4	395 (41.8)	1.18 (0.99, 1.40)	95 (10.1)	1.28 (0.89, 1.83)	300 (31.7)	1.15 (0.94, 1.41)
≤14	122,895	7.0	336 (47.8)	1.29 (1.07, 1.55)**	77 (11.0)	1.35 (0.92, 1.97)	259 (36.9)	1.27 (1.03, 1.57)*
Unknown	77,687	4.1	178 (43.5)	1.23 (1.00, 1.52)*	44 (10.7)	1.32 (0.86, 2.01)	134 (32.7)	1.21 (0.95, 1.54)
P for trend				<0.001		<0.01		<0.01
Total	778,334	48.2	1,827 (37.9)		423 (8.8)		1,404 (29.1)	

Fig. S5. The association between baseline dental health condition and the risk of gastric cancer among individuals aged >70 years old in the Swedish Dental Health Register, 2009-2016. All HR and 95% CI estimates were derived from Cox models with attained age as time-scale, adjusted for sex, age at entry, family income, education, family history of gastric cancer, smoking-related diseases, and alcohol-related diseases. Trend analyses were performed by Cochran-Armitage test. The 'unknown' group was excluded from the calculation of *p*-trend. Abbreviations: IR, incidence rate; HR, hazard ratio; CI, confidence interval. *: p < 0.05; **: p < 0.01; ***: p < 0.001.

Group	No. of subjects	Person years (× 100,000)	All gastric cancer: No. of cases (IR, per 100,000 person years)	All gastric cancer: Fully-adjusted HR (95% CI)	Cardia gastric cancer: No. of cases (IR, per 100,000 person years)	Cardia gastric cancer: Fully-adjusted HR (95% CI)	Non-cardia gastric cancer: No. of cases (IR, per 100,000 person years)	Non-cardia gastric cancer: Fully-adjusted HR (95% CI)
Dental health condition								
Healthy	2,687,218	180.2	1,167 (6.5)	Ref.	364 (2.0)	Ref.	803 (4.5)	Ref.
Caries	911,409	61.5	470 (7.6)	1.16 (1.04, 1.30)**	160 (2.6)	1.28 (1.05, 1.55)*	310 (5.0)	1.11 (0.96, 1.27)
Root canal infection	245,120	15.9	143 (9.0)	1.25 (1.05, 1.50)*	54 (3.4)	1.38 (1.02, 1.87)*	89 (5.6)	1.19 (0.95, 1.50)
Mild inflammation	1,019,675	68.2	547 (8.0)	1.13 (1.02, 1.26)*	162 (2.4)	1.08 (0.89, 1.31)	385 (5.6)	1.16 (1.02, 1.32)*
Periodontitis	648,012	44.4	566 (12.7)	1.16 (1.04, 1.29)**	202 (4.5)	1.33 (1.11, 1.60)**	364 (8.2)	1.08 (0.95, 1.23)
P for trend				<0.01		<0.05		0.068
Number of teeth at baseline								
>27	2,962,028	197.8	758 (3.8)	Ref.	279 (1.4)	Ref.	479 (2.4)	Ref.
>24 & ≤27	916,863	64.6	617 (9.6)	1.09 (0.97, 1.22)	202 (3.1)	1.04 (0.86, 1.27)	415 (6.4)	1.12 (0.97, 1.29)
>20 & ≤24	458,175	32.0	531 (16.6)	1.28 (1.13, 1.45)***	161 (5.0)	1.27 (1.02, 1.57)*	370 (11.5)	1.29 (1.10, 1.50)**
>14 & ≤20	241,507	16.4	407 (24.8)	1.50 (1.30, 1.73)***	130 (7.9)	1.67 (1.31, 2.13)***	277 (16.9)	1.44 (1.21, 1.71)***
≤14	170,425	11.1	305 (27.6)	1.65 (1.41, 1.92)***	81 (7.3)	1.63 (1.23, 2.16)***	224 (20.2)	1.66 (1.38, 2.00)***
Unknown	762,436	48.3	275 (5.7)	1.33 (1.15, 1.55)***	89 (1.8)	1.29 (1.00, 1.67)*	186 (3.9)	1.35 (1.13, 1.63)**
P for trend				<0.001		<0.01		<0.001
Total	5,511,434	370.2	2,893 (7.8)		942 (2.5)		1,951 (5.3)	

Low risk ← → High Risk

Fig. S6. The association between baseline dental health condition and the risk of gastric cancer after excluding incident cases and follow-up data observed within the first two years. All HR and 95% CI estimates were derived from Cox models with attained age as time-scale, adjusted for sex, age at entry, family income, education, family history of gastric cancer, smoking-related diseases, and alcohol-related diseases. Trend analyses were performed by Cochran-Armitage test. The 'unknown' group was excluded from the calculation of *p*-trend. Abbreviations: IR, incidence rate; HR, hazard ratio; CI, confidence interval. *: $p < 0.05$; **: $p < 0.01$; ***: $p < 0.001$.

Group	No. of subjects	Person years (× 100,000)	All gastric cancer: No. of cases (IR, per 100,000 person years)	All gastric cancer: HR (95% CI)	Cardia gastric cancer: No. of cases (IR, per 100,000 person years)	Cardia gastric cancer: HR (95% CI)	Non-cardia gastric cancer: No. of cases (IR, per 100,000 person years)	Non-cardia gastric cancer: HR (95% CI)
Dental health condition								
Healthy	2,320,826	149.8	1,327 (8.9)	Ref.	394 (2.6)	Ref.	933 (6.2)	Ref.
Caries	812,991	52.9	531 (10.0)	1.08 (0.97, 1.20)	163 (3.1)	1.11 (0.92, 1.34)	368 (7.0)	1.07 (0.94, 1.21)
Root canal infection	194,432	12.0	140 (11.7)	1.18 (0.99, 1.41)	46 (3.8)	1.22 (0.89, 1.66)	94 (7.8)	1.16 (0.94, 1.44)
Mild inflammation	921,271	58.7	610 (10.4)	1.07 (0.96, 1.18)	184 (3.1)	1.03 (0.86, 1.24)	426 (7.3)	1.08 (0.96, 1.22)
Periodontitis	573,213	37.8	636 (16.8)	1.13 (1.03, 1.25)*	215 (5.7)	1.29 (1.09, 1.54)**	421 (11.1)	1.06 (0.94, 1.20)
P for trend				<0.05		<0.05		0.195
Number of teeth at baseline								
>27	3,094,328	195.5	924 (4.7)	Ref.	322 (1.6)	Ref.	602 (3.1)	Ref.
>24 & ≤27	894,857	61.2	760 (12.4)	1.05 (0.95, 1.17)	233 (3.8)	1.06 (0.88, 1.27)	527 (8.6)	1.05 (0.93, 1.20)
>20 & ≤24	436,600	29.6	656 (22.2)	1.22 (1.09, 1.37)***	188 (6.4)	1.37 (1.12, 1.69)**	468 (15.8)	1.16 (1.01, 1.34)*
>14 & ≤20	230,159	14.9	518 (34.6)	1.43 (1.25, 1.62)***	155 (10.4)	1.79 (1.42, 2.25)***	363 (24.3)	1.30 (1.11, 1.52)***
≤14	166,789	10.1	386 (38.2)	1.44 (1.25, 1.66)***	104 (10.3)	1.71 (1.31, 2.22)***	282 (27.9)	1.34 (1.13, 1.59)***
P for trend				<0.001		<0.001		<0.001
Total	4,822,733	311.2	3,244 (10.4)		1,002 (3.2)		2,242 (7.2)	

Fig. S7. The association between baseline dental health condition and the risk of gastric cancer after excluding participants with missing covariate data. All HR and 95% CI estimates were derived from Cox models with attained age as time-scale, adjusted for sex, age at entry, family income, education, family history of gastric cancer, smoking-related diseases, and alcohol-related diseases. Trend analyses were performed by Cochran-Armitage test. The 'unknown' group was excluded from the calculation of *p*-trend. Abbreviations: IR, incidence rate; HR, hazard ratio; CI, confidence interval. *: $p < 0.05$; **: $p < 0.01$; ***: $p < 0.001$.
